# A Simple Clinical Pre-procedure Risk Model for Predicting Thrombocytopenia Associated With Periprocedural Use of Tirofiban in Patients Undergoing Percutaneous Coronary Intervention

**DOI:** 10.3389/fphar.2018.01456

**Published:** 2018-12-10

**Authors:** Yi-Hu Yi, Wen-Jun Yin, Zhi-Chun Gu, Wei-Jin Fang, Dai-Yang Li, Can Hu, Kun Liu, Rong-Rong Ma, Ling-Yun Zhou

**Affiliations:** ^1^Department of Pharmacy, Third Xiangya Hospital, Central South University, Changsha, China; ^2^School of Xiangya Medical Science, Central South University, Changsha, China; ^3^Department of Pharmacy, Renji Hospital, Shanghai Jiao Tong University, Shanghai, China; ^4^College of Pharmacy, Xinjiang Medical University, Xinjiang, China

**Keywords:** thrombocytopenia, tirofiban, risk scores, prediction model, percutaneous coronary intervention

## Abstract

**Background:** No risk model for predicting thrombocytopenia associated with periprocedural tirofiban exposure is available. The purpose of this study was to develop a simple clinical pre-procedure risk model based on pre-procedural characteristics for early prediction of thrombocytopenia before patients were exposed to tirofiban.

**Methods:** The series included 1862 patients who underwent percutaneous coronary intervention with tirofiban exposure. Baseline demographic and clinical characteristics were collected from the hospital information system on admission. The earliest pro-procedural platelets within 72 h were used to evaluate the thrombocytopenia incidence. Risk factors associated with thrombocytopenia in patients with tirofiban exposure were investigated by univariable and multivariable analyses. Locally weighted scatterplot smoothing procedure was used to identify the cut points for the numeric variables. The discriminatory power of the scoring system was assessed with the receiver operating characteristic (ROC) curve analysis.

**Results:** The occurrence of thrombocytopenia was 4.02% (75 of 1862), 4.01% (56 of 1396), and 4.08% (19 of 466) in the overall, developmental, and validation data sets, respectively. The risk score was developed based on five independent predictors: age ≥65y, white blood cell ≥12 × 10^9^/L, diabetes mellitus, congestive heart failure, and chronic kidney disease. This tool was well calibrated (Hosmer Lemeshow χ^2^ = 6.914; *P* = 0.546) and good discrimination was well obtained in validation data set (C-statistic, 0.82).

**Conclusion:** The clinical pre-procedure risk model is a simple and accurate tool for early identification of high-risk patients of thrombocytopenia before tirofiban exposure, allowing for timely and appropriate intervention.

## Introduction

Tirofiban, as a Glycoprotein IIb/IIIa (GP IIa/IIIb) receptor antagonist (GPRA), can inhibit the platelet aggregation through binding to the GP IIb/IIIa receptor, and then reduce the risk of ischemic events in patients undergoing percutaneous coronary interventions (PCI; Kondo and Umemura, 2002). Previous clinical trials have confirmed that tirofiban can reduce the occurrence of death, myocardial infarction and refractory ischemia events compared with control treatment (Platelet Receptor Inhibition in Ischemic Syndrome Management Study Investigators, 1998; Platelet Receptor Inhibition in Ischemic Syndrome Management in Patients Limited by Unstable Signs and Symptoms Study Investigators, 1998; Van’t-Hof et al., 2008).

Although widely regarded as safe, tirofiban has been reported to be associated with thrombocytopenia based on several case reports, case series and clinical trials with incidence ranging from 0.4 to 5.6% (Merlini et al., 2004; Mulot et al., 2004; Demirkan et al., 2006; Sakellariou et al., 2009). The underlying mechanism of tirofiban-associated thrombocytopenia has not been completely understood. Some researchers found it might be associated with immune-mediated reactions (Merlini et al., 2004). Lower platelet counts associated with acute and severe thrombocytopenia in a patient may led to the increased risk for serious bleeding and mortality, during or shortly after tirofiban exposure (Merlini et al., 2004). In the *post hoc* analysis from PRISM trial, thrombocytopenia was associated with a 5- to 10- fold increased risk for bleeding complications (Adamo et al., 2016). Thus, early, timely and specific interventions, such as changing anticoagulation strategies, may be required in patients with severe thrombocytopenia.

However, monitoring post-procedural platelet counts are not regularly performed in patients with tirofiban exposure in clinical practice for increased clinical costs, especially in middle-income regions (Ibanez et al., 2018). Furthermore, no research has been performed to investigate the risk factors of tirofiban-associated thrombocytopenia. Thus, it is difficult for clinicians to identify patient who is at high risk for thrombocytopenia. Even the patient who has developed thrombocytopenia may not be noticed until the severe bleeding complications occur, which are associated with prolonged in-hospital stay, and increased health care costs, morbidity, even mortality. Accordingly, identifying patients at high risk of thrombocytopenia for early and timely intervention may be essential.

Thus, we conducted the present study to investigate the risk factors of tirofiban-associated thrombocytopenia and to develop a simple clinical identification tool that is available for pre-tirofiban exposure prediction of thrombocytopenia in patients undergoing PCI.

## Materials and Methods

### Ethics Statement

We performed a retrospective study in hospitalized patients at Third Xiangya Hospital of Central South University, Changsha, China, from September 2007 to December 2017. The study protocol was approved by the Medical Ethical Committee in the Xiangya Hospital of Central South University (No: 2017-S275). Written consents given by the patients were waived as the data used in this study were anonymized.

### Study Subjects

Patients were identified by the electronic medical record system (EMRs) of the Third Xiangya Hospital and enrolled if they were treated with tirofiban during and shortly after the PCI procedure as guidelines recommended. All the patients undergoing PCI, whether elective PCI or urgent PCI, were enrolled in the present study, including those with acute coronary syndromes and/or chronic stale CAD. The index date was defined as the date of initial prescription of tirofiban. Patients were excluded for platelet counts <150 × 10^9^/L at the time of screening or without platelet counts within 30 days before tirofiban treatment or 72 h after treatment with tirofiban (Platelet Receptor Inhibition in Ischemic Syndrome Management in Patients Limited by Unstable Signs and Symptoms Study Investigators, 1998). Also, patients with prescription for GRPAs during the preceding 3 months from the index date, or with 4T score >3 points were excluded. All patients received antiplatelet or anticoagulation therapy if it was required by their medical conditions as guideline recommended. Tirofiban was administered in the event of angiographic evidence of a large thrombus, slow- or no- reflow, and other thrombotic complications for up to 18 h. Intravenous unfractionated heparin (UFH) was routinely used in almost all patients during the PCI procedure, except for those who have received prior LMWH treatment.

### Platelet Monitoring and Clinical Definitions

Latest platelet counts within 30 days before tirofiban treatment was defined as the baseline platelet counts, and the earliest platelet counts within 72 h after the procedure was used to evaluate the thrombocytopenia incidence. Thrombocytopenia was defined as a platelet count of <100 × 10^9^/L within 72 h of tirofiban exposure (Huxtable et al., 2006). Mild and severe thrombocytopenia were defined as platelet counts 50–100 × 10^9^/L and <50 × 10^9^/L (Huxtable et al., 2006). 4T pretest probability score was used to assess the probability of heparin induced thrombocytopenia (HIT) according to the degree and timing of thrombocytopenia, the presence of thrombosis, and the likelihood of other causes of thrombocytopenia: high probability (6–8 points), intermediate probability (4–5 points), and low probability (≤3 points) (Linkins et al., 2013). The 4T score was determined by two independent clinicians, with adjudication by a third physician researcher in the case of discrepancy. Co-morbidities were identified by both International Classification of Diseases, 10 Revision, coding (ICD 10) and detailed clinical information recorded. Besides the diagnostic information recorded in the hospital EMRs, diabetes mellitus (DM) was also defined as fasting blood glucose >126 mg/dL, postprandial plasma glucose >200 mg/dL, glycated hemoglobin >6.5%, or the use of antidiabetic medications; hypertension (HP) was defined as repeated measurements of blood pressure (BP) >140/90 mmHg or on treatment with antihypertensive medications; congestive heart failure was defined as New York Heart Association functional class III-IV for the clinical implication.

### Covariates

To identify patients who are at high risk of tirofiban- associated thrombocytopenia, baseline demographic and clinical characteristics were collected from the hospital EMRs on admission. All the data were reviewed by two independent clinicians.

### Statistical Analysis

Baseline continuous data was presented as mean ± SD. Categorical variables were presented as proportion. Eligible patients were randomly assigned to developmental and validation data set in 3:1 manner, respectively. Developmental data set was used to identify the independent risk factors associated with thrombocytopenia. Candidate predictors that were significant in univariable analysis (*P* < 0.05) or of clinical importance were included in the multivariable analysis. Then, backward logistic regression was used to identify the independent predictors of thrombocytopenia and to estimate the odds ratios (Ors). Variables with a significant level of 0.05 in the model or with known clinical important could stay in the final model.

Then, a risk scoring system is devised from the results of the multivariable analysis. The method was similar to that of Jean-Roger Le-Gall et al (1996). First step, we used the locally weighted scatterplot smoothing (LOWESS) procedure to identify the cut points of the continuous variables that would define the ranges of predictive ability for each variable. Second step, dummy variables were created for each range, and then were entered into a multiple logistic regression model to calculate associated coefficient (β). Third step, all the β’s for variables were grouped by the different levels of increasing probability levels of thrombocytopenia based on the observed range of the coefficients, and a new dummy value for each level of probability of thrombocytopenia was defined. Lastly, we calculated the score by summing the points of each risk factor for each patient. The summed point of each patient was then used as variable in a logistic regression equation: logit = β0 + β1(score). The logit was then converted to a probability of thrombocytopenia as P(y = 1| logit) = e^logit^/(1 + e^logit^), where P indicated probability, y equaled 1 for patients who was developed to thrombocytopenia, y equaled 0 for patients who was not.

The risk scoring system was tested in the validation data set. Goodness of fit of the scoring system was evaluated with the Hosmer–Lemeshow test. The discriminatory power was assessed with the calculation of the area under the receiver operating characteristic (ROC) curve (AUC).

Statistical analyses were carried out using R V.3.3.0^1^.

## Results

### Data Collection and Clinical Characteristics

**FIGURE 1:**
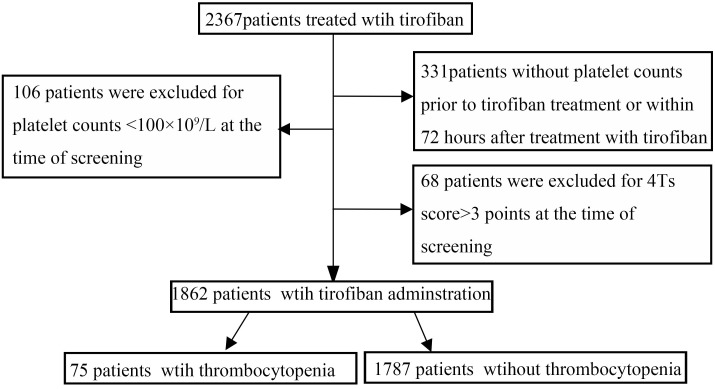
Flow chart depicting numbers of patients who were included in analysis after exclusion criteria. The total included encounters were divided into those with and without tirofiban-induced thrombocytopenia.

Of a total of 2367 patients treated with tirofiban, 492 patients were excluded due to exclusion criteria. One thousand, eight hundred sixty-two patients with platelet counts at baseline and 72 h within the procedure were enrolled in this study as shown in Figure 1. Of them, 75 (4.02%) developed thrombocytopenia: mild in 59 (78.6%) and severe in 16 (21.4%). Among the 1862 patients, 56 (4.01%) experienced thrombocytopenia in the developmental data set, whereas 19 of 466 (4.07%) experienced thrombocytopenia in the validation data set. UFH (1423 of 1862, 76.6%) was demonstrated to be more widely used anticoagulant in our PCI setting compared with LMWH (439 of 1862, 23.4%) in combination with tirofiban. Seventy-nine variables including demographic information, co-morbidities, concomitant drugs, and laboratory values were collected as baseline demographic and clinical characteristics for each patient (Table 1).

**Table 1:** Baseline demographic characteristics of patients with and without thrombocytopenia in developmental data set.

Variable	Thrombocytopenia (*n* = 56)	No thrombocytopenia (*n* = 1340)
**Demographic information**		
Men	14(25.0%)	356(26.6%)
Age, y	62.78 ± 10.93	60.66 ± 10.87
Age ≥ 65y	29(51.8%)	518(38.66%)
Height (cm)	164.89 ± 6.55	165.0 ± 7.00
Weight (kg)	65.89 ± 9.68	65.4 ± 10.6
**Co-morbidities**		
Hypertension	35(62.5%)	786(58.7%)
Diabetes mellitus	37(66.1%)	627(46.8%)
Stable angina	5(9%)	49(3.7%)
Unstable angina	10(17.8%)	382(28.5%)
Myocardial infarction	41(73.2%)	909(67.8%)
Urgent PCI procedure	19(33.9%)	424(31.6%)
Cerebral infarction	9(16.1%)	191(14.2%)
Dyslipidemia	14(25.0%)	257(19.2%)
Congestive heart failure (NYHAIII/IV)	30(53.6%)	441(32.9%)
Heart failure with NYHA I/II	5(8.9%)	136(10.1%)
Infectious diseases	19(33.9%)	409(30.5%)
Chronic kidney disease	12(21.4%)	143(10.7%)
Liver dysfunction	19(33.9%)	436(32.5%)
Peripheral vascular disease	3(5.35%)	64(4.78%)
**Laboratory values**		
White blood cell (×10^9^/L)	12.26 ± 5.54	9.34 ± 3.75
Red blood cell (×10^12^/L)	4.26 ± 0.63	4.39 ± 0.57
Hematocrit (%)	39.27 ± 5.99	40.42 ± 4.79
Hemoglobin (g/L)	127.58 ± 20.75	133.03 ± 17.02
Mean corpuscular hemoglobin (pg)	30.02 ± 2.97	30.39 ± 2.37
Platelet counts (×10^9^/L)	208.79 ± 58.09	204.36 ± 54.23
Plateletocrit (%)	0.23 ± 0.11	0.22 ± 0.05
Mean platelet volume (fL)	10.98 ± 1.45	10.97 ± 1.41
Platelet distribution width (fL)	15.87 ± 1.99	15.64 ± 2.13
Monocytes (×10^9^/L)	0.53 ± 0.29	0.52 ± 0.27
Monocytes%	5.64 ± 2.21	5.99 ± 2.45
Lymphocytes (×10^9^/L)	1.67 ± 0.82	1.65 ± 0.79
Lymphocytes %	19.35 ± 9.99	17.72 ± 9.81
Neutrophil (×10^9^/L)	7.89 ± 5.33	7.03 ± 3.71
Neutrophil %	73.19 ± 11.96	72.54 ± 11.83
Basophil (×10^9^/L)	0.03 ± 0.02	0.02 ± 0.02
Basophil %	0.32 ± 0.25	0.30 ± 0.25
Eosinophils (×10^9^/L)	0.11 ± 0.11	0.10 ± 0.14
Eosinophils %	1.44 ± 1.80	1.36 ± 1.77
Albumin (g/L)	38.49 ± 4.23	39.24 ± 4.44
Alanine aminotrans (U/L)	92.13 ± 374.41	44.49 ± 82.01
Glutamic-oxalacetic transaminase (U/L)	200.13 ± 583.33	112.40 ± 160.06
Total bilirubin (μmol/L)	14.34 ± 6.39	15.06 ± 6.54
Direct bilirubin (μmol/L)	4.77 ± 3.51	4.52 ± 2.61
Total bile acids (μmol/L)	3.93 ± 4.72	3.56 ± 4.06
Total protein (g/L)	63.55 ± 6.10	64.70 ± 6.33
Globulin (g/L)	25.08 ± 4.45	25.46 ± 4.07
Urea (mmol/L)	5.93 ± 2.89	5.03 ± 1.92
Uric acid (μmol/L)	329.27 ± 116.38	313.54 ± 103.99
Creatinine (μmol/L)	90.91 ± 47.07	81.90 ± 29.42
Triglycerides (mmol/L)	1.63 ± 1.00	1.74 ± 1.12
Total cholesterol (mmol/L)	4.42 ± 1.26	4.68 ± 1.12
High density lipoprotein (mmol/L)	1.10 ± 0.26	1.18 ± 0.29
Low density lipoprotein (mmol/L)	2.45 ± 0.88	2.62 ± 0.88
Fasting blood glucose (mmol/L)	7.89 ± 4.44	6.95 ± 4.59
Myoglobin (ng/mL)	302.09 ± 370.83	293.37 ± 363.01
Creatine kinase isoenzyme (U/L)	85.53 ± 148.14	74.31 ± 111.27
Lactate Dehydrogenase (U/L)	405.38 ± 371.71	344.56 ± 208.20
Creatine kinase (U/L)	837.90 ± 1363.18	660.41 ± 997.81
TnI (ng/mL)	5.14 ± 5.21	6.78 ± 27.90
α-Hydroxybutyrate Dehydrogenase (U/L)	345.99 ± 249.41	318.15 ± 194.82
Sodium (mmol/L)	140.09 ± 3.38	139.77 ± 3.29
Potassium (mmol/L)	4.19 ± 0.47	4.09 ± 0.46
Chlorine (mmol/L)	103.29 ± 4.04	103.64 ± 3.71
Calcium (mmol/L)	2.23 ± 0.16	2.26 ± 0.18
Thrombin time (sec)	23.32 ± 22.41	23.00 ± 23.27
International normalized ratio	1.08 ± 0.24	1.03 ± 0.18
Prothrombin time (sec)	12.55 ± 1.99	12.37 ± 2.04
Fibrinogen (g/L)	3.53 ± 1.39	3.42 ± 1.06
D-dimer (mg/L)	236.29 ± 528.32	178.04 ± 431.19
Brain natriuretic peptide (pg/mL)	2708.58 ± 3138.79	1766.15 ± 1782.07
Ejection fraction (%)	53.75 ± 10.65	57.88 ± 9.67
**Concomitant drugs**		
Angiotensin receptor blockers	5(8.93%)	125(9.33%)
Angiotension converting enzyme inhibitors	36(64.29%)	904(67.46%)
βblocker	47(80.36%)	1010(75.37%)
Diuretic	25(44.64%)	321(23.96%)
Calcium channel blockers	13(23.21%)	374(27.91%)
Statins	53(94.64%)	1212(90.45%)
Proton Pump Inhibitor	49(87.50%)	1185(88.43%)
Aspirin (75–100 mg/d)	56(100%)	1340(100%)
ADP receptor antagonist	56(100%)	1340(100%)
Insulin	30(53.57%)	510(38.06%)
Oral antidiabetic medications	15(26.79%)	252(18.80%)
Glucocorticoids	8(14.29%)	130(9.70%)
Heparin	45(80.4%)	1034(77.2%)
Low molecular weight heparin	11(19.6%)	306(22.8%)


### Risk Factors for Thrombocytopenia Associated With Tirofiban

In order to investigate the risk factors of thrombocytopenia, the univariable analysis was performed. A total of 21 variables were significantly associated with the development of thrombocytopenia, including demographics (age), co-morbidities (DM, congestive heart failure, and chronic kidney disease), several laboratory values (white blood cell, red blood cell, hematocrit, hemoglobin, neutrophil, urea, creatinine, total cholesterol, high density lipoprotein, low density lipoprotein, fasting blood glucose, potassium, international normalized ratio, ejection fraction) and several concomitant drugs (β-blocker, diuretic, and insulin, Table 2). Treatment with UFH or LMWH was found to be not associated with the development of thrombocytopenia after excluding those with 4T score >3 points.

**Table 2:** Univariate and multivariable logistic regression analysis of risk factors that were selected to develop the risk model for predicting thrombocytopenia (developmental data set).

Variable	Univariable Analysis			Multivariable Analysis		
		
	OR	95%CI	*P* Value	OR	95%CI	*P*
Age	2.18	1.23–2.13	<0.001	2.03	1.38–3.28	0.001
Diabetes mellitus	2.09	1.38–3.22	<0.001	2.01	1.29–3.15	0.003
Congestive heart failure	2.26	1.49–3.41	<0.001	1.51	1.47–2.38	0.032
Chronic kidney disease	2.22	1.29–3.74	0.003	1.45	1.12–2.61	0.041
White blood cell	2.19	1.45–3.31	<0.001	1.41	1.24–2.26	0.027
Red blood cell	0.68	0.48–0.97	0.035			
Hematocrit	0.96	0.92–0.99	0.028			
Hemoglobin	0.98	0.97–0.99	0.004			
Neutrophil	1.05	1.01–1.09	0.042			
Urea	1.18	1.08–1.30	<0.001			
Creatinine	1.01	1.01–1.03	0.018			
Total cholesterol	0.81	0.66–0.97	0.028			
High density lipoprotein	0.38	0.17–0.79	0.011			
Low density lipoprotein	0.79	0.62–1.01	0.068			
Fasting blood-glucose	1.03	0.99–1.03	0.074			
Potassium	1.12	1.11–1.68	0.037			
International normalized ratio	2.84	1.13–7.17	0.024			
Ejection fraction	0.96	0.94–0.98	<0.001			
βblocker	1.62	0.97–2.83	0.077			
Diuretic	2.47	1.61–3.76	<0.001			
Insulin	1.86	1.24–2.81	0.003			


After considering both of the statistical significance and the clinical implication, 11 variables, including age, white blood cell, hematocrit, creatinine, total cholesterol, fasting blood glucose, ejection fraction, DM, congestive heart failure, chronic kidney disease, and treatment with diuretic were enrolled for the conduction of a backward multivariable analysis to increase the ratio of events per variable (EPV). And finally, age, white blood cell, DM, congestive heart failure and chronic kidney disease were identified as independent predictors in the final model. And no significant 2–way interaction was founded (Table 2). The results were confirmed by selecting the different variables for the multivariable analysis (Supplementary Table S1).

### Risk Score Development

For scoring purpose, age and white blood cell were divided into categories regarding predictive ability, with the cutoff of 65 years and 12 × 10^9^/L, respectively, by LOWESS method. The score points for each range of each variable was created as shown in Table 3. Patients with a higher risk score presented a higher probability of developing thrombocytopenia. The total risk score ranges from a minimum value of 0 to a maximum value of 8, with predicted probabilities of thrombocytopenia ranging from 0.56 to 17.1%.

**Table 3:** Scores points for the independent variables.

Risk factors	Points
**Age**	
≥65y	2
50–65y	0
**White blood cell**	
≥12 × 10^9^/L	1
<12 × 10^9^/L	0
**Diabetes mellitus**	
Yes	2
No	0
**Congestive Heart failure**	
Yes	2
No	0
**Chronic kidney disease**	
Yes	1
No	0


### Clinical Implications of the Risk Score Model

**FIGURE 2:**
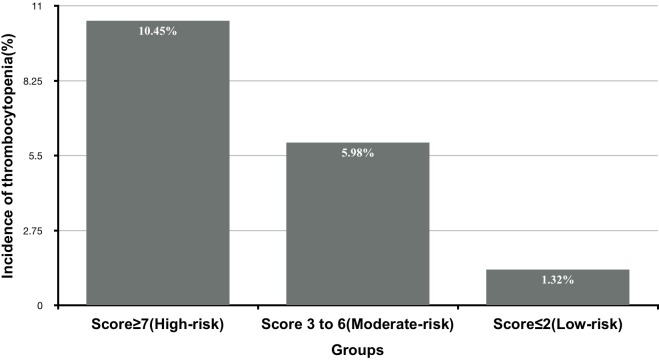
The incidence of thrombocytopenia according to different levels of risk score derived from development data set.

Based on the obtained frequencies of thrombocytopenia in relation to different risk scores, 1396 patients were further categorized into three levels to enhance the clinical utility of the risk score model: low-risk [*n* = 430 (30.8%)], moderate-risk [*n* = 628 (44.9%)], and high-risk [*n* = 282 (20.2%)], corresponding to risk scores of ≤2, 3–6, and ≥7, respectively (Figure 2).

### Discrimination and Calibration of the Risk Score in the Validation Data Set

**FIGURE 3:**
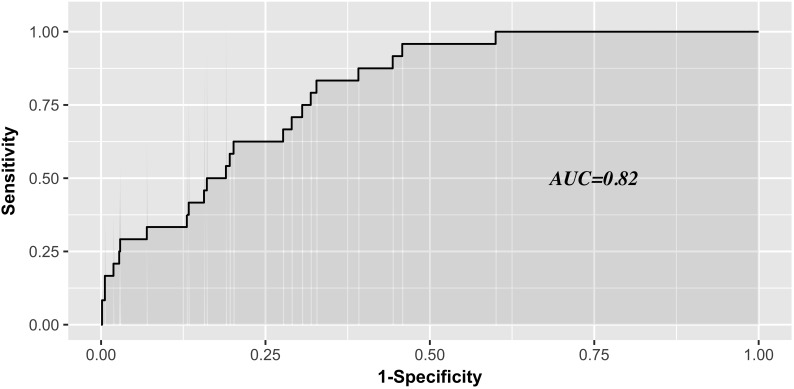
Receiver Operating characteristics analysis—area under the curve according to risk score in the validation series.

**FIGURE 4:**
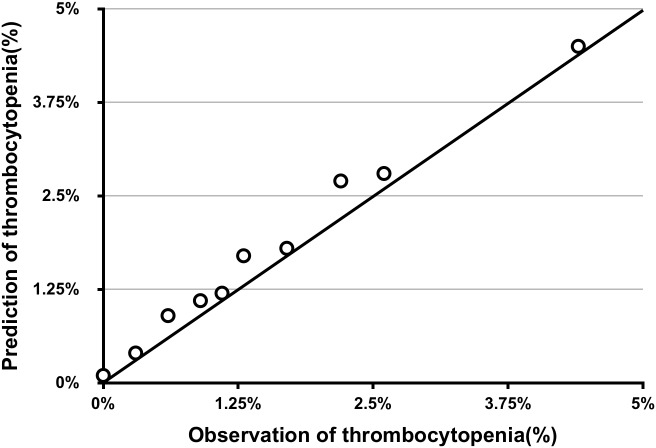
Calibration plot of predicted vs. observed thrombocytopenia of risk score in the validation series.

The AUC for the predictive scoring system was 0.82 (Figure 3) in the validation data set. In the total patient population, the predicted probability of thrombocytopenia according to the risk score was 5.66%. Figure 4 reports the calibration plot of the risk score in the validation dataset. Overall, there is a good calibration for predicted probability of thrombocytopenia.

## Discussion

To our knowledge, this is the first study to investigate the risk factors of thrombocytopenia associated with peri-procedural tirofiban exposure in patients undergoing PCI. Furthermore, we developed a simple and accurate clinical pre-procedure risk model to assist clinicians to early identify high-risk patients before tirofiban exposure, allowing for timely risk allocation and appropriate intervention.

Previous clinical trials have demonstrated that tirofiban can reduce the risk of ischemic events by inhibiting the platelet GP Iib/IIIa receptor (Platelet Receptor Inhibition in Ischemic Syndrome Management in Patients Limited by Unstable Signs and Symptoms Study Investigators, 1998). Based on these results, present guidelines have recommended that GPRAs should be considered for bailout if there is evidence of no-reflow or a thrombotic complication (Ibanez et al., 2018). However, the increased use of GPRAs has reported to be associated with thrombocytopenia, with the incidence ranging from 0.5 to 5.6% (Le-Gall et al., 1996; Dasgupta et al., 2000; Llevadot et al., 2000; Tcheng, 2000; Topol et al., 2001; Bougie et al., 2002). In our study, the incidence of thrombocytopenia in patients with tirofiban exposure was 4.02% in the total data set, which is similar to previous reports.

Besides tirofiban, there are some other medications which can induce thrombocytopenia too. The best well-known one is heparin. Anticoagulation with heparin is used to prevent ischemic complications secondary to plaque disruption and endothelial injury during PCI and was also recommended by guidelines (Ibanez et al., 2018). There are two types of HIT. Heparin therapy causes platelet aggregation, which can lead to a transient, mild drop in platelet counts 48–72 h after initiation of heparin therapy, which is known as HIT 1 type. HIT type II is an adverse immune-mediated drug reaction that is associated with a high risk of venous and arterial thrombosis (Greinacher et al., 2008; Warkentin et al., 2010). For some common characteristics, it’s difficult to distinguish the HIT and tirofiban-associated thrombocytopenia in clinical practice. The most convenient method to evaluate the probability of HIT is 4T score which is based on the history and physical examination (Linkins et al., 2013). According to the score model, 6–8 points, 4–5 points, and ≤3 points indicate high probability, intermediate probability and low probability, respectively. Patients with 4T score ≥4 points were recommended to detect antibodies against PF4/heparin to definitely diagnose HIT (Linkins et al., 2013). In present study, we ruled out 68 patients with 4T score ≥4 points to make the results more reliable to predict the probability of tirofiban associated thrombocytopenia. However, for the limitation of retrospective clinical trial, we couldn’t detect the antibodies of these patients enrolled in or ruled out, which might still affect the prediction ability of developed risk tool on some extent. Besides heparin, anti-platelet medications, such as aspirin and clopidogrel, have also been reported to be associated with thrombocytopenia (Hu et al., 2013). But none of the 56 patients developed thrombocytopenia discontinued dual anti-platelet therapy in the in-hospital period after clearly checking the EMRs by two independent clinicians. Thus, we considered thrombocytopenia of those patients as tirofiban-associated thrombocytopenia.

Thrombocytopenia associated bleeding complications may induce prolonged hospital stay, increased morbidity or mortality and unfavorable health care costs (Merlini et al., 2004; Adamo et al., 2016). However, monitoring post-procedural platelet counts are not regularly performed in clinical practice for patients with tirofiban exposure for increased clinical costs, especially in middle-income regions (Ibanez et al., 2018). Thus, it is difficult for the clinicians to timely identify high-risk patients and make early intervention for the patients who have developed thrombocytopenia. Until now, the limited evidences of the association between tirofiban exposure and thrombocytopenia are mainly based on the case reports or *post-hoc* analysis of several clinical trials. And, the risk factors of thrombocytopenia in patients with tirofiban exposure have not been examined systematically. Thus, one of the objects of present study was to investigate the risk factors of tirofiban-associated thrombocytopenia, and then develop a simple and accurate predictive tool for clinical assessment. According to univariable and multivariable analysis, five independent risk factors of tirofiban-associated thrombocytopenia were identified from 79 baseline clinical characteristics, including age ≥65y, white blood cell ≥12 × 10^9^/L, DM, congestive heart failure and chronic kidney disease.

Age ≥65y, DM and congestive heart failure were awarded the two points, respectively. Age ≥65y is a known common factor to evaluate the ischemic and bleeding risks for the special physiological status of the old patient. In our study, we also found that patients developed thrombocytopenia were older than patients without thrombocytopenia (62.78 ± 10.93 vs. 60.66 ± 10.87). Multiple mechanisms have been suggested to play a role in the dysfunction of platelets in DM patients. However, most of the previous clinical trials and mechanism investigations focused on the platelet hyper-reactivity, increased ischemic events and endothelial dysfunction in DM patients (Ferreiro et al., 2010). Only two clinical studies reported the higher risk of thrombocytopenia in DM patients. A study (Chen et al., 2015) in Taiwan concluded that patients with DM tended to increase person’s susceptibility to thrombocytopenia. The other (Kapoor et al., 2015) reported that presence of DM was a predictor of intracranial bleeding and thrombocytopenia in elderly patients. In our present study, we found that 664 patients (47.6%) had the co-morbidity of DM. Furthermore, patients with DM had a higher frequency of thrombocytopenia (5.51% vs. 2.66%). Congestive heart failure, which we defined as NYHA functional class III/IV for the clinical implication, was the other important risk factor in the scoring system. In previous studies (Kandis et al., 2011; Kaya et al., 2017), it has been shown that mean platelet volume can be elevated in heart failure patients, predicting high risk of thrombotic events. On the other hand, thrombocytopenia and bleeding were also reported as clinical complications in heart failure patients. Palva et al. (1970) found thrombocytopenia in 6% of their heart failure patients. In our series, the incidence of thrombocytopenia was 6.37% in congestive heart failure patients, similar to previous report. Most recently, Mondal et al. (2017a,b) (Haro et al., 2000) reported that oxidative stress induced modulation of platelet integrin α2bβ3 expression and shedding may play a potential role in the platelet apoptosis, inducing thrombocytopenia and non-surgical bleeding among heart failure patients.

Chronic kidney disease and white blood cell ≥12 × 10^9^/L were awarded the one point, respectively. Chronic kidney disease patients have high risks of both bleeding and thrombosis (Fox et al., 2010). However, no clinical trial has been performed to directly investigate the association between thrombocytopenia and chronic kidney disease. The present study observed that 11.1% patients were co-morbidity of chronic kidney disease, and thrombocytopenia was probably more common in patients with chronic kidney disease than in patients with normal renal function (7.74% vs. 3.28%). White blood cells are the cells of the immune system that are involved in protecting the body against both infectious disease and foreign invaders. Fountain et al. (2017) found that of the 533 patients diagnosed with clostridium difficile infection (CDI) patients, with a high level of white blood cell, moderate thrombocytopenia (platelet counts <100 × 10^9^/L at time of CDI diagnosis) was present in 15% of the patients. Consistent with previous study, our univariable analysis revealed that white blood cell was the independent risk factor of thrombocytopenia in patients treated with tirofiban. Furthermore, the cut point of 12 × 10^9^/L was identified by LOWESS procedure. Thus, in the scoring system, patients with the level of white blood cell >12 × 10^9^/L was given one score. Recently, it is recognized that infection can predispose to the formation of immune complexes resulting in thrombocytopenia (Poskitt and Poskitt, 1985). Although, the increase of white blood cells in circulation is most commonly caused by infection, whether the association between high level of white blood cell and thrombocytopenia results from a generalized inflammatory response or is directly attributable to the microorganism requires further study.

Although, the predictive discriminations and calibrations of the predictive scoring system are excellent, there are some limitations of this study. The retrospective nature is the primary limitation of present study by possible selection and information bias of data collected in hospital EMRs. However, the main endpoint assessment of present study was mostly relied on the accurate record keeping, such as laboratory values, instead of subjective indicators, such as the patient’s individual recall of former exposure to risk variables which may be inaccurate and subject to biases. Furthermore, we collected almost all the data recorded in the hospital EMRs, including demographic, information, co-morbidities, concomitant drugs, and laboratory values for each patient to alleviate the information bias. Additionally, retrospective nature of present study didn’t allow us to collect the blood samples of patients enrolled in the cohort to perform the laboratory testing to make a definite HIT diagnosis, which might affect the results on some extent. Although, we have excluded patients with 4Ts score ≥4, a large prospective study is really needed to validate our data, shed additional light on this area. The second limitation is that we exclude almost half of the patients with tirofiban exposure because of the lack of post-procedural platelet counts. That may induce a higher frequency of the thrombocytopenia than the actual conditions. The third limitation is that the small size of the thrombocytopenia patients. The reported frequency of the thrombocytopenia in patients with tirofiban exposure is 0.5∼5.6%, and the frequency is 4.02% (56 of 1396 patients) in our series. Because of the small size of the target patients, some variables, such as gender, were not significant upon multivariable modeling. Furthermore, there may be indeed insufficient power to determine higher dimensional models in the scoring system. However, this is a common problem to the existing scoring systems to predict the risk of adverse drug reaction or clinical outcomes with low frequency (Gatti et al., 2017). To solve this problem, larger sample cohort should be needed in further investigation. The fourth limitation is lack of the comparison of the performance with other predictive models. No other predictive model or scoring system has been published before. Although, the scoring system performed well in our data set, further external validation is really needed to confirm the results or modify the model. Additionally, this study did not consider some potentially important factors contributed to the risk of thrombocytopenia during the procedure, such as the time of the procedure and the type of the stent, because of the too many missing data.

## Conclusion

This scoring system derived from real-world populations to predict drug associated adverse events is simple and accurate. This model is not only to aid the clinicians identifying the high-risk patients who need special care, but also to avoid the unnecessary regular monitoring of the platelet counts in low-risk patients with tirofiban exposure. However, further larger validation studies are essential before introducing the scoring system into the clinical practice.

## Author Contributions

Y-HY and L-YZ conceived and designed the study. W-JY, KL, CH, D-YL, and R-RM acquired the data. Z-CG, W-JF, Y-HY, and L-YZ analyzed and interpreted the data. Y-HY and L-YZ drafted the manuscript. L-YZ critically revised the manuscript.

## Conflict of Interest Statement

The authors declare that the research was conducted in the absence of any commercial or financial relationships that could be construed as a potential conflict of interest.

## Footnotes

1 http://www.R-project.org/
