# Supplementary material for: A Simple Clinical Pre-procedure Risk Model for Predicting Thrombocytopenia Associated With Periprocedural Use of Tirofiban in Patients Undergoing Percutaneous Coronary Intervention
Source: Front Pharmacol. 2018 Dec 10;9:1456. doi: 10.3389/fphar.2018.01456 (PMC6295459; doi:10.3389/fphar.2018.01456)
Supplement: Supplementary file 1 [file Table_1.docx]

**SUPPLEMENTAL MATERIAL**

**A simple clinical pre-procedure risk model for predicting thrombocytopenia associated with periprocedural use of tirofiban in patients undergoing percutaneous coronary intervention**

Yi-hu Yi^a,b^, Wen-jun Yin^a^, Zhi-chun Gu^c^, Wei-jin Fang^a^, Dai-yang Li^a,b^, Can Hu^a,b^, Kun Liu^a,b^, Rong-rong Ma^d^, Ling-yun Zhou^a,*^

**Online Contents**

Supplementary Results………………………………………………………………………….………..…2

Supplementary Appendix 1. Multivariable logistic regression analysis of risk factors that were selected to develop the risk model for predicting thrombocytopenia (developmental data set)…….……………….2

**Supplementary Methods**

**Supplementary Appendix 1. Multivariable logistic regression analysis of risk factors that were selected to develop the risk model for predicting thrombocytopenia (developmental data set)**

Supple.Table 1. Univariate and multivariable logistic regression analysis of risk factors that were selected to develop the risk model for predicting thrombocytopenia (developmental data set)

| Variable | Univariable Analysis | | | Multivariable Analysis | | |
| --- | --- | --- | --- | --- | --- | --- |
|  | OR | 95%CI | *P* Value | OR | 95%CI | *P* |
| Age | 2.18 | 1.23-2.13 | <0.001 | 2.11 | 1.42-3.57 | 0.000 |
| Diabetes mellitus | 2.09 | 1.38-3.22 | <0.001 | 1.95 | 1.33-2.86 | 0.001 |
| Congestive heart failure | 2.26 | 1.49-3.41 | <0.001 | 1.72 | 1.27-2.79 | 0.041 |
| Chronic kidney disease | 2.22 | 1.29-3.74 | 0.003 | 1.53 | 1.22-2.58 | 0.037 |
| White blood cell | 2.19 | 1.45-3.31 | <0.001 | 1.28 | 1.11-2.34 | 0.031 |
| Hematocrit | 0.96 | 0.92-0.99 | 0.028 |  |  |  |
| Creatinine | 1.01 | 1.01-1.03 | 0.018 |  |  |  |
| Total cholesterol | 0.81 | 0.66-0.97 | 0.028 |  |  |  |
| Fasting blood-glucose | 1.03 | 0.99-1.03 | 0.074 |  |  |  |
| βblocker | 1.62 | 0.97-2.83 | 0.077 |  |  |  |
| Diuretic | 2.47 | 1.61-3.76 | <0.001 |  |  |  |
| Insulin | 1.86 | 1.24-2.81 | 0.003 |  |  |  |
